# Comparative Evaluation of the Antimicrobial Activity of Different Antimicrobial Peptides against a Range of Pathogenic Bacteria

**DOI:** 10.1371/journal.pone.0144611

**Published:** 2015-12-11

**Authors:** Anna Ebbensgaard, Hanne Mordhorst, Michael Toft Overgaard, Claus Gyrup Nielsen, Frank Møller Aarestrup, Egon Bech Hansen

**Affiliations:** 1 National Food Institute, Technical University of Denmark, Søltofts Plads, 2800 Kgs, Lyngby, Denmark; 2 National Food Institute, Technical University of Denmark, Mørkhøj Bygade 19, 2860, Søborg, Denmark; 3 Department of Chemistry and Bioscience, Aalborg University, Fredrik Bajers Vej 7H, 9220, Aalborg, Denmark; Centre National de la Recherche, FRANCE

## Abstract

**Analysis of a Selected Set of Antimicrobial Peptides:**

The rapid emergence of resistance to classical antibiotics has increased the interest in novel antimicrobial compounds. Antimicrobial peptides (AMPs) represent an attractive alternative to classical antibiotics and a number of different studies have reported antimicrobial activity data of various AMPs, but there is only limited comparative data available. The mode of action for many AMPs is largely unknown even though several models have suggested that the lipopolysaccharides (LPS) play a crucial role in the attraction and attachment of the AMP to the bacterial membrane in Gram-negative bacteria. We compared the potency of Cap18, Cap11, Cap11-1-18m^2^, Cecropin P1, Cecropin B, Bac2A, Bac2A-NH_2_, Sub5-NH_2_, Indolicidin, Melittin, Myxinidin, Myxinidin-NH_2_, Pyrrhocoricin, Apidaecin and Metalnikowin I towards *Staphylococcus aureus*, *Enterococcus faecalis*, *Pseudomonas aeruginosa*, *Escherichia coli*, *Aeromonas salmonicida*, *Listeria monocytogenes*, *Campylobacter jejuni*, *Flavobacterium psychrophilum*, *Salmonella typhimurium* and *Yersinia ruckeri* by minimal inhibitory concentration (MIC) determinations. Additional characteristics such as cytotoxicity, thermo and protease stability were measured and compared among the different peptides. Further, the antimicrobial activity of a selection of cationic AMPs was investigated in various *E*. *coli* LPS mutants.

**Cap18 Shows a High Broad Spectrum Antimicrobial Activity:**

Of all the tested AMPs, Cap18 showed the most efficient antimicrobial activity, in particular against Gram-negative bacteria. In addition, Cap18 is highly thermostable and showed no cytotoxic effect in a hemolytic assay, measured at the concentration used. However, Cap18 is, as most of the tested AMPs, sensitive to proteolytic digestion *in vitro*. Thus, Cap18 is an excellent candidate for further development into practical use; however, modifications that should reduce the protease sensitivity would be needed. In addition, our findings from analyzing LPS mutant strains suggest that the core oligosaccharide of the LPS molecule is not essential for the antimicrobial activity of cationic AMPs, but in fact has a protective role against AMPs.

## Introduction

The extensive use of classical antibiotics not only in human medicine, but also in animal farming for treatment and growth promotion has resulted in the development and spread of antibiotic resistance in bacteria. There is now an increasing awareness of the problems for human health caused by antibiotic resistant bacteria among food producing animals, especially since many of the same classes of antibiotics are used in both reservoirs [1]. This has emphasized the need for new solutions to battle infections in farmed animals which are not based on antibiotics that are considered critically important for human health [2].

Antimicrobial peptides (AMPs) represent an attractive alternative to classical antibiotics. AMPs are present in all kingdoms of life and are ancient components of the innate immunity and represent the first line of defense in an infection [3,4]. Despite their diversity in sequence, they generally have an overall positive charge (+2 to +9) and have a substantial proportion of hydrophobic amino acids (= >30%). The length normally varies between 10–50 amino acids. Based on these properties, AMPs are able to fold into amphiphilic three-dimensional structures, which are divided into 4 major groups: α-helical peptides (e.g. Cecropin B, Cecropin P1, Melittin, Cap11, Cap18 and magainins); β-sheet peptides with 2–4 disulfide bridges (e.g. human defensins, plectasin or protegrins); extended peptides which are rich in glycin, proline, tryptophan, and/or histidine (e.g. Indolicidin, Apidaecin); loop peptides with one disulfide bridge (e.g. bactenecin). The majority of the so far characterized AMPs belong to the group of the α-helical peptides and the β-sheet peptides [3][5][6]. AMPs isolated from prokaryotes are called bacteriocins. One of the best characterized bacteriocins is Nisin, which is originally isolated from *Lactococcus lactis*. Nisin is active against various major Gram-positive food-borne pathogens, including *Listeria* and *Clostridium*. It is used as food additive and preservative since 1969 in processed cheese, pasteurized milk and milk products and cooked sausages [7].

Classically, the mechanisms of AMP action are thought of as an interaction with the bacterial cell membrane. Most often, the interaction of an AMP with the membrane will lead to destabilization of the membrane by formation of transient channels, micellarization, and dissolution of the membrane or translocation across the membrane which results in increased membrane permeability. As a result of increased permeability, nutrients and electrolytes will flow out which lead to killing of the bacteria. However, killing might also happen in a more specific or targeted manner by recognition of cell surface proteins as a first step followed by insertion in the membrane. Finally, the mode of action might also be targeting metabolic processes in the bacteria including cell wall synthesis, nucleic acid or protein synthesis, which are vital to the organism [8].

Several studies have reported activity of different peptides useful for food preservation and safety purposes [7][9][10][11]. However, most of these studies have only focused on a single or a few peptides and a single or a few bacterial targets. Thus, a major bottleneck in identifying which new AMPs to choose for further development into practical use is the lack of comprehensive overviews comparing the potency of known AMPs from different sources to a broader range of pathogens. In addition, cytotoxicity and stability is most often ignored, although being crucial parameters in developing successful AMP alternatives.

In this study, we focused on finding AMPs to inhibit zoonotic and fish pathogens. Aquaculture is a high density animal production system characterized by a high use of antimicrobial agents. For this study we have selected a handful of peptides reported to have high antibacterial activity against Gram-negative bacteria. In addition we required the peptides to be composed of ordinary L-amino acids, devoid of posttranslational modifications, and to be shorter than 40 aa. These selection criteria were chosen to allow for subsequent development of recombinant production procedures for peptides with potential applications in food or feed. We compared the potency of the selected AMPs towards a broad range of pathogens under standardized and comparable conditions with respect to antimicrobial activity, hemolytic activity, and stability. In addition, the mode of action of the most potent AMPs has been addressed in the *E*. *coli* ATCC25922 which was chosen as model organism. The present work will facilitate the evaluation and identification of AMPs for further development.

## Materials and Methods

### Bacterial Strains and Growth Conditions

The strains used in this study are listed in Table 1. The *Yersinia ruckeri* strain was kindly provided by Prof. Kurt Buchmann, University of Copenhagen, Faculty of Health and Medical Sciences, and the *Flavobacterium psychrophilum* strain was kindly provided by Prof. Inger Dalsgaard, DTU, Denmark. BW25113 is the *Escherichia coli* wild-type strain, a derivative of the F^-^,λ^-^ *E*. *coli* K12 strain BD792 which was used in generating the KEIO collection [12][13]. *E*. *coli* ATCC25922 is a clinical isolate, serotype O6 and is often used as control strain in antimicrobial susceptibility testing. All strains were grown in Mueller-Hinton-II medium, except *L*. *monocytogenes* which was grown in BHI medium and *F*. *psychrophilum* which was grown in TYES medium (Tryptone yeast extract plus salts [14]). Incubation took place aerobically at 37°C, except for *Y*. *ruckeri* and *A*. *salmonicida*, which were grown aerobically at RT (20°C), *C*. *jejuni* NCTC11168 which was grown under microaerophilic conditions at 42°C and *F*. *psychrophilum* 1947 which was grown under aerobic conditions at 15°C. All plates were incubated for 18–20 hours, except the *F*. *psychrophilum* plates, which were incubated for 72 hours.

**Table 1:** Strains used in this study.

Strain	Relevant characteristics /genotype	Reference(s)
*Staphyloccous aureus* ATCC29213	control strain for antimicrobial susceptibility testing	ATCC strain collection
*Enterococcus faecalis* ATCC29212	control strain for antimicrobial susceptibility testing	ATCC strain collection
*Pseudomonas aeruginosa* ATCC27853	control strain for antimicrobial susceptibility testing	ATCC strain collection
*Escherichia coli* ATCC25922	Clinical isolate, Serotype O6, Biotype 1, control strain for antimicrobial susceptibility testing	ATCC strain collection
*Aeromonas salmonicida* ATCC33658	Type strain	ATCC strain collection
*Salmonella enterica serovar* Typhimurium LT2		sequenced strain
*Listeria monocytogenes* N22-2	Isolate from fish processing industry	[58]
*Campylobacter jejuni* NCTC11168	Isolate from human feces	NCTC strain collection
*Flavobacterium psychrophilum* 1947		Prof. Inger Dalsgaard, DTU, Denmark
*Yersinia ruckeri* 392/2003		[59]
*Escherichia coli* BW25113	F^-^, Δ(*araD-araB*)*567*, Δ*lacZ4787(*::*rrnB-3)*, λ^-^, *rph-1*, Δ(*rhaD-rhaB*)*568*, *hsdR514*, wild-type strain used in the KEIO collection	[12][13]
*Escherichia coli* JW3596	BW25113 *rfaC*::*kan*	[13]
*Escherichia coli* JW3024	BW25113 *rfaE*::*kan*	[13]
*Escherichia coli* JW3595	BW25113 *rfaF*::*kan*	[13]
*Escherichia coli* JW3606	BW25113 *rfaG*::*kan*	[13]
AD120	*Escherichia coli* ATCC25922 Δ*rfaC*	This study
AD121	*Escherichia coli* ATCC25922 Δ*rfaE*	This study
AD122	*Escherichia coli* ATCC25922 Δ*rfaF*	This study
AD123	*Escherichia coli* ATCC25922 Δ*rfaF*	This study

### Antimicrobial peptides

The peptides used in this study are listed in Table 2. Cecropin P1 and Cecropin B were purchased from Sigma-Aldrich. Cecropin P1 with a purity of ≥95% and Cecropin B with a purity of ≥97% and were dissolved in water. Bac2A-NH_2_ with a purity of ≥95% and Sub5-NH_2_ with a purity ≥95% were purchased from Anaspec (distributed by BioNordika Denmark A/S). The peptide sequences were confirmed with MS data. Bac2A-NH_2_ was dissolved in 100% DMSO, whereas Sub5-NH_2_ was dissolved in water. Cap18 (89.5% purity, 58% net peptide content), Cap11 (94.7% purity, 63% net peptide content), Cap11-1-18m^2^ (87.9% purity, 57% net peptide content), Bac2A (93.2% purity), Myxinidin (97% purity) and Myxindin-NH_2_ (97.3% purity, 66% net peptide content) were all synthesized at Genscript. Bac2A, Cap18, Cap11-1-18m2, Myxinidin and Myxinidin-NH_2_ were dissolved in water and Cap11 was dissolved in 100% DMSO. Melittin (RP10290-1) and Indolicidin (RP11242-0.5) each with a purity of >95% were purchased from Genscript and dissolved in water. The proline rich peptides, Pyrrhocoricin, Apidaecin IA and Metalnikowin I, were purchased from Anaspec each with a purity of ≥95% and dissolved in water. All peptides were dissolved to a stock concentration of 10 mg/ml.

**Table 2:** Sequence and origin of antimicrobial peptides.

Peptide	Sequence	Origin	Structure	Reference
**Cap11**	GLRKKFRKTRKRIQKLGRKIGKTGRKVWKAWREYGQIPYPCRI	mammalian, guinea pig, neutrophils	α-helical	[29]
**Cap11-1-18m** ^**2**^	KLRKLFRKLLKLIRKLLR	truncated derivative of Cap11	α-helical	[21]
**Cap18**	GLRKRLRKFRNKIKEKLKKIGQKIQGLLPKLAPRTDY	mammalian, rabbit, neutrophils	α-helical	[28][30]
**Cecropin P1**	SWLSKTAKKLENSAKKRISEGIAIAIQGGPR	mammalian, pig, small intestine	α-helical	[32][33][34]
**Cecropin B**	KWKVFKKIEKMGRNIRNGIVKAGPAIAVLGEAKALG-NH_2_	insects, giant silk moth, pupae	α-helical	[33][35]
**Bac2A**	RLARIVVIRVAR	non-amidated version of Bac2A-NH_2_	α-helical/β-sheet	
**Bac2A-NH**_**2**_	RLARIVVIRVAR-NH_2_	linear variant of Bactenecin from bovine neutrophils	Linearized version of bactenecin	[36][37]
**Sub5-NH**_**2**_	RRWKIVVIRWRR-NH_2_	synthetic variant of Bac2A-NH_2_	Not available	[36]
**Myxinidin**	GIHDILKYGKPS	fish, epidermal mucus of Hagfish	Not available	[38]
**Myxinidin-NH**_**2**_	GIHDILKYGKPS-NH_2_	amidated form of myxinidin	Not available	
**Pyrrhocoricin**	VDKGSYLPRPTPPRPIYNRN	insects, Pyrrhocoris apterus	Not available	[20]
**Apidaecin IA**	GNNRPVYIPQPRPPHPRI	insects, honey bee	Extended, proline rich	[60]
**Metalnikowin I**	VDKPDYRPRPRPPNM	insects, palomena prasina	Not available	[19]
**Melittin**	GIGAVLKVLTTGLPALISWIKRKRQQ-NH_2_	insects, honey bee	α-helical	[16]
**Indolicidin**	ILPWKWPWWPWR-NH_2_	mammalian, bovine neutrophils	Extended	[17]

### Antimicrobial susceptibility testing (MIC testing)

The minimum inhibitory concentrations (MICs) of the AMPs were measured in 96-well microtiter plates according to the Clinical and Laboratory Standards Institute (CLSI, formerly National Committee for Clinical Laboratory Standards [NCCLS]) [15]. Briefly, liquid Mueller-Hinton-II medium containing increasing concentrations of AMPs is inoculated with a defined number of cells (approx. 10^5^ CFUs/ml) in 96-well microtiter plates (polypropylene), whereas each plate also includes a positive growth control and a negative control (sterile control). After incubation, the MIC is determined by the lowest concentration showing no visible growth. All plates were incubated for 18–20 hours, except the *F*. *psychrophilum* plates which were incubated for 72 hours.

All the MIC measurements were carried out in duplicate. The MIC of the reference antibiotics was determined by the use of Sensititre panels (Trek Diagnostic Systems Ltd, East Grinstead, UK).

### Cytotoxicity assay

The cytotoxicity for each AMP was determined spectrophotometrically by measuring the haemoglobin release from horse erythrocytes. Briefly, fresh defibrinated horse blood was washed three times with PBS, centrifuged for 15 minutes at 1000g and resuspended at 10% (v/v) in PBS. Samples of the washed horse erythrocytes (100 μl) were transferred to a 96 well microtiter plate and mixed with 100 μl AMP solution. PBS was used as a negative control, and 0.2% TritonX-100 was used as a positive control. The microtiter plates were incubated for 60 minutes at 37°C and then centrifuged for 10 minutes at 1300g. The supernatants were transferred to a flat-bottom 96 well polystyrene microtiter plate and the haemoglobin release was monitored by measuring the absorbance at 540 nm. The percentage of hemolysis was calculated as 100 *(A_sample_−A_PBS_)/(A_TritonX-100_ –A_PBS_), where A_sample_ is the experimental absorbance of the peptide sample, A_PBS_ is the control absorbance of untreated erythrocytes, and A_TritonX-100_ is the absorbance of 0.2% TritonX-100 lysed cells.

### Effect of temperature and proteases on antimicrobial activity

AMPs were heated at 70°C or 90° for 5, 15 or 30 minutes. An untreated control, which was kept at RT, was used as a control. After incubation at 70°C or 90°C, the minimum inhibitory concentrations (MICs) of the peptides were measured in 96-well microtiter plates according the Clinical and Laboratory Standards Institute (CLSI, formerly National Committee for Clinical Laboratory Standards [NCCLS]) [15] (see under *Antimicrobial susceptibility testing (MIC testing)*. *E*. *coli* ATCC25922 was used as a test strain for all the AMPs. The effect of proteases, including trypsin (trypsin ultra, NEB, P8101) and proteinase K (NEB, P8107S), on antimicrobial activity of selected AMPs was investigated by incubation with the respective protease at 37°C for either 30 seconds, 2, 5, 15 or 30 minutes. The protease to AMP ratio used in the assay was 1:100 (w/w) and the buffers used were trypsin-ultra reaction buffer (NEB, P8101) for trypsin and 100 mM Tris-HCl pH 7.5 for proteinase K digestion. To ensure that the trypsin or proteinase K themselves have no antimicrobial activity, each of the protease was used alone in the corresponding buffer as a control. After incubation, the samples were cooked for 10 minutes at 90°C to inactivate the protease. Afterwards, the antimicrobial activity was determined by measuring the minimal inhibitory concentration (see Antimicrobial susceptibility testing)). *E*. *coli* ATCC25922 was used as a test strain.

### Construction of LPS mutants of *E*. *coli* ATCC25922

Construction of knock-out mutants of *E*. *coli* ATCC25922 were constructed using the λ-Red recombinase gene replacement system [12]. Primers for amplification of the *npt* gene of pKD4 are listed in S1 Table. The correct double-crossover and recombination event was confirmed by primers listed in S1 Table and by sequencing. Finally, the kanamycin cassette was removed [12].

## Results

### Antimicrobial activity of selected antimicrobial peptides

Fifteen antimicrobial peptides from different classes and origins (Table 2), including the well-characterized AMPs Melittin [16] and Indoclidin [17], were selected and tested for antimicrobial activity. The antimicrobial activity was determined against 10 different bacterial strains from 10 different species (Table 1) as the minimum inhibitory concentration (MIC), summarized in Table 3. In addition, standard antibiotics from different classes were included for the comparison of the antimicrobial activity. No activity against *F*. *psychrophilum* could be measured, whereas a varying pattern of activity was found against the other bacterial species tested. Cap18 had the highest antimicrobial activity of all tested AMPs, in particular against Gram-negative pathogens, whereas the other cathelicidin, Cap11, was slightly less active. The antimicrobial activity of both cathelicidins was in general higher against Gram-negative compared to Gram-positive bacteria. In contrast, Cap11-1-18m^2^, which is a short derivative of Cap11, had only moderate antimicrobial activity against Gram-negative pathogens, but increased antimicrobial activity against Gram-positive bacteria compared to the mother peptide Cap11. Interestingly, Cap11-1-18m^2^ displayed the same high specific activity against *C*. *jejuni* as full length Cap11. Cecropin P1 and Cecropin B showed specific antimicrobial activity against Gram-negative bacteria, whereas no activity was detected against any of the Gram-positive bacteria tested. Cecropin B had moderate antimicrobial activity against all tested Gram-negative pathogens, while Cecropin P1 had specific activity against *Y*. *ruckeri*, *A*. *salmonicida* and *E*. *coli* only. Bac2A and its amidated form Bac2A-NH_2_ are linear C2A/C11A variants of the naturally occurring bovine peptide bactenecin. Bac2A displayed very low antimicrobial activity against the tested microorganisms except for *E*. *faecalis* and *L*. *monocytogenes*. Sub5-NH_2_, a synthetic derivative of Bac2A-NH_2_ carrying five mutations, had strongly increased antimicrobial activity compared to the mother peptide Bac2A-NH_2_. No difference in the specificity between gram-negative and gram-positive organisms was observed for the bactenectin-derived peptides. The well-characterized peptide Melittin has good activity against gram-positive bacteria; in particular *S*. *aureus*, *E*. *faecalis* and *L*. *monocytogenes*. Indolicidin shows only moderate activity except for *L*. *monocytogenes*. Myxinidin, originally isolated from the epidermal mucus of the hagfish, and it´s amidated form Myxinidin-NH_2_ displayed no antimicrobial activity under any of the tested conditions. Pyrrhocoricin, Apidaecin IA and Metalnikowin I are all belonging to the family of the glycine-rich peptides [18][19][20]. Only Apidaecin showed specific antimicrobial activity against *S*. *enterica* serovar Typhimurium and *E*. *coli*, whereas the other AMPs were ineffective (MIC ≥ 256 μg/ml). The potency of the AMPs was compared to the antimicrobial activity of well-characterized antibiotics from different classes either targeting the bacterial cell wall, the protein or nucleic acid synthesis. The MIC values for the reference antibiotics are summarized in Table 3. The solvent DMSO alone had no antimicrobial activity in the concentration range used in the assay (data not shown).

**Table 3:** Antimicrobial activities of antimicrobial peptides and antibiotics against Gram-negative and Gram-positive bacteria.

	*Y. ruckeri* 392/2003	*A. salmonicida* ATCC33658	*F. psychrophilum* 1947	*S*. *enterica* ser. Typhimurium LT2	*C. jejuni* NCTC11168	*E*. *coli* ATCC25922	*P. aeruginosa* ATCC27853	*S. aureus* ATCC29213	*E. faecalis* ATCC29212	*L. monocytogenes* N22-2
**Antimicrobial Peptides**										
**Cap18**	2	2	>256	4	1	4–8	4–8	≥32	8	2–4
**Cap11**	4	4	>256	8	4	8–16	8	16–32	16–32	32
**Cap11-1-18m** ^**2**^	8–16	32–64	>256	16	2	16–32	16–32	16	8–16	16
**Cecropin P1**	32	64	>256	≥128	>256	16–32	>256	>256	>256	>256
**Cecropin B**	32	32–64	>256	32	16	16–32	64	>256	>256	>256
**Bac2A-NH**_**2**_	≥256	128	>256	128	64–128	64	128–256	128	16–32	8
**Bac2A**	256	>256	>256	>256	≥256	256	256	>256	64	32
**Sub5-NH**_**2**_	16–32	8	>256	8	8	4	8	8	4–8	2
**Myxinidin**	>256	>256	>256	>256	>256	>256	>256	>256	>256	>256
**Melittin**	16–32	>64	n.d.	32–64	2–4	16	≥64	2–4	2–4	2–4
**Indolicidin**	≥64	≥64	n.d.	64	16	32	>64	32	32	4
**Myxinidin-NH**_**2**_	>256	>256	>256	>256	>256	>256	>256	>256	>256	>256
**Pyrrhocoricin**	>256	>256	n.d.	>256	n.d.	>256	>256	>256	>256	>256
**Apidaecin IA**	256	>256	n.d.	64	n.d.	32	>256	>256	>256	>256
**Metalnikowin I**	>256	>256	n.d.	>256	n.d.	>256	>256	>256	>256	>256
**Antibiotics**										
**Ampicillin**	2	≤1	n.d.	≤1	4	4	>64	≤1	≤1	≤1
**Gentamicin**	1–2	1	n.d.	0.5–1	≤0.5	1	1–2	≤0.5	8–16	≤0.5
**Colisitin (Polymyxin E)**	1–2	2	n.d.	2	16	≤1	2–4	>16	>16	>16
**Azithromycin**	≤2	≤2	n.d.	4	≤2	4	>64	≤2	4	≤2
**Cefotaxime**	≤0.25	≤0.25	n.d.	≤0.25	≥4	≤0.25	>4	2	>4	>4
**Chloramphenicol**	≤8	≤8	n.d.	≤8	≤8	≤8	≥128	≤8	≤8	≤8
**Ciprofloxacin**	0.06	≤0.015	n.d.	≤0.015	0.12	≤0.015	0.25	0.25	0.5	1
**Meropenem**	≤0.03	≤0.03	n.d.	≤0.03	≤0.03	≤0.03	0.25	0.12	4	0.12
**Nalidixic acid**	32	≤4	n.d.	≤4	≤4	≤4	64	64	≥128	≥128
**Ceftaxidime**	≤0.5	≤0.5	n.d.	≤0.5	>8	≤0.5	2	8	>8	>8
**Tetracycline**	≤2	≤2	n.d.	≤2	≤2	≤2	16	≤2	16	≤2
**Tigecycline**	≤0.25	≤0.25	n.d.	≤0.25	≤0.25	≤0.25	4	≤0.25	≤0.25	≤0.25
**Trimethoprim**	1	≤0.25	n.d.	≤0.25	>32	0.5	>32	2	≤0.25	≤0.25

Data are collected as minimal inhibitory concentrations (MICs) according to the Clinical and Laboratory Standards Institute (CLSI) and expressed in μml. All MIC determinations were carried out in duplicates for AMPs and in triplicate for antibiotics; n.d. = not determined.

### Hemolytic activity

Not only the antimicrobial activity, but also the ability to differentiate between bacterial and mammalian cells is an important factor for a successful antimicrobial peptide. The cytotoxicity of the selected AMPs was therefore determined using a hemolytic assay based on lysis of washed horse erythrocytes. A minimal peptide concentration of 64 μg/ml, which was above or in the MIC range for the corresponding AMPs, and a maximum peptide concentration of 256 μg/ml, limited by the experimental setup, was used. Melittin showed a very high hemolytic activity at the peptide concentration of 128 μg/ml (110% compared to the 0.1% Trition X-100 control), Cap11-l-18m^2^ showed a significant hemolytic activity (52% compared to the triton X-100 control) at the peptide concentration of 64 μg/ml and indolicidin was slightly hemolytic (12% compared to the 0.1% trition X-100 control at the peptide concentration of 128 mg/ml), whereas the other tested peptides had no to minimal hemolytic activity indicating that they might be safe to use in the concentrations tested (Table 4). The solvent DMSO alone had no hemolytic activity in the concentration range used in the assay (data not shown).

**Table 4:** Hemolytic activities of the antimicrobial peptides against horse erythrocytes.

Peptide	Peptide Concentration [μg/ml]	Hemolytic Activity[%]*
Cap11	64	4 ± 0
Cap11-1-18m^2^	64	52 ± 6
Cap18	64	1 ± 0
Melittin	128	110 ± 1
Indolicidin	128	12 ± 0
Cecropin P1	256	0 ± 0
Cecropin B	256	0 ± 0
Bac2A	256	0 ± 0
Bac2A-NH_2_	256	0 ± 0
Sub5-NH_2_	256	0 ± 0
Myxinidin	256	0 ± 0
Myxinidin-NH_2_	256	0 ± 0
Pyrrhocoricin	256	0 ± 0
Apidaecin IA	256	0 ± 0
Metalnikowin I	256	0 ± 0

* The hemolytic activity is measured in duplicates and given as the average ± SD in % relative to full lysis induced by 0.2% Triton X-100.

### Thermostability and protease stability

To address the question of stability, the thermostability and protease stability against the commercial available proteases trypsin and proteinase K was investigated. Only AMPs with high antimicrobial activity (MIC ≤ 32 μg/ml for at least two bacterial species) were included in the stability assays. *E*. *coli* ATCC25922 was used as test strain for both, the thermostability and protease stability assays.

The thermostability was measured by the determination of the antimicrobial activity of the peptides after incubation for 5, 15 and 30 minutes at 70°C or 90°C. All the tested peptides retained their antimicrobial activity even after incubation at 70°C or 90°C for either 5, 15 or 30 minutes (Table 5). All the tested AMPs are stable at high temperatures.

**Table 5:** Thermostability of antimicrobial peptides.

Temperature									
	Incubation Time[min]								
		Antimicrobial Activity MIC [μg/ml]							
		Cap18	Cap11	Cap11-1-18m^2^	Cecropin B	Cecropin P1	Melittin	Indolicidin	Sub5
70°C	0	8	8	32	16	16	16	32	4
	5	8	8	32	16	16	16	32	4
	15	8	8	32	16	16	16	32	4
	30	8	8	32	16	16	16	32	8
90°C	0	8	8	32	16	16	16	32	4
	5	8	8	32	16	16	16	32	4
	15	8	8	32	16	16	16	32	4
	30	8	8	32	16	16	16	32	4

Data are collected as minimal inhibitory concentrations (MICs) according to the Clinical and Laboratory Standards Institute (CLSI) and expressed in μml. All MIC values are the average of five independent experiments.

The remaining antimicrobial activity after incubation with either trypsin or proteinase K for different incubation times is summarized in Table 6. The antimicrobial activity of Cecropin B, Cecropin P1and Melittin is completely abolished after a very short incubation of only 30 seconds with proteinase K. Cap18 and Indolicidin show a two-fold decreased antimicrobial activity after incubation with proteinase K for 2 minutes; respectively 4-fold decreased antimicrobial activity after incubation for 30 minutes. Sub5 shows a similar pattern with a 2-fold reduced activity after 30 sec, a 4-fold reduction after 2 minutes, an 8-fold reduction after 5 minutes, and a 16-fold reduction after 15 or 30 minutes. Interestingly, Cap11-1-18m^2^ showed a 2-fold increased antimicrobial activity after very short incubation of 30 seconds or 2 minutes with proteinase K. However, incubation of more than 15 minutes with proteinase K leads to the complete loss of antimicrobial activity of Cap11-1-18m^2^.

**Table 6:** Protease stability of antimicrobial peptides.

Protease										
	Incubation Time[min]									
			Antimicrobial Activity MIC [μg/ml]							
		Cap18	Cap11	Cap11-1-18m^2^	Cecropin B	Cecropin P1	Melittin	Indolicidin	Sub5	Gentamicin
Proteinase K	0	8	16	16	16	16	16	32	4	1
	0.5	8	16	8	>64	>64	>64	32	8	1
	2	16	16	8	>64	>64	>64	32	16	1
	5	16	32	16	>64	>64	>64	64	32	1
	15	32	64	>64	>64	>64	>64	64	64	1
	30	32	>64	>64	>64	>64	>64	64	64	1
Trypsin	0	8	8	16	16	32	16	32	4	1
	0.5	>64	16	8	>64	>64	>64	>64	4	1
	2	>64	32	8	>64	>64	>64	>64	8	1
	5	>64	64	8	>64	>64	>64	>64	8	1
	15	>64	>64	32	>64	>64	>64	>64	16	1
	30	>64	>64	>64	>64	>64	>64	>64	64	1

Data are collected as minimal inhibitory concentrations (MICs) after incubation with proteinase K or trypsin according to the Clinical and Laboratory Standards Institute (CLSI) and expressed in μml. All MIC determinations were carried out in triplicates.

The incubation of Cap18, Cecropin P1, Cecropin B, Melittin and Indolicidin with trypsin lead to a complete loss of antimicrobial activity after only 30 seconds of incubation. For Cap11, the incubation with trypsin resulted in a 2-fold decrease of antimicrobial activity after 30 seconds, a 4-fold decrease after 2 minutes, 8-fold decrease after 5 minutes and a complete loss of activity after 15, respectively 30 minutes. Sub5 followed a similar pattern; incubation with trypsin reduced the antimicrobial activity by factor 2 after 2 and 5 minutes, by factor 4 after 15 minutes incubation and by factor 16 after 30 minutes incubation. A short incubation with trypsin of up to 5 minutes increased the antimicrobial activity of Cap11-1-18m^2^ by factor 2. Incubation times longer than 5 minutes lead to a 2-fold reduction, while 15 minutes of incubation decreases the activity by factor 2, incubation times longer than 30 minutes are leading to a complete loss of antimicrobial activity. In contrast to thermostability, the majority of the tested AMPs were highly sensitive towards the proteases trypsin and proteinase K. The antibiotic gentamicin was used as a control, which retained its full antimicrobial activity after incubation with trypsin or proteinase K for 30 minutes.

### Lipopolysaccharides are not target for the AMPs

**Fig 1:**
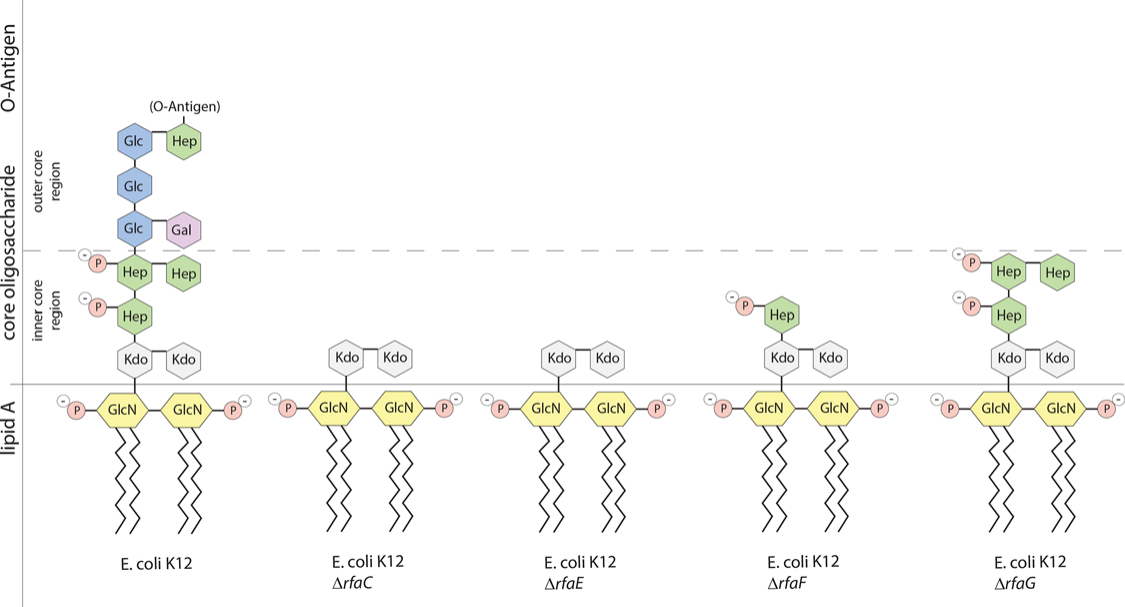
Schematic lipopolysaccharide structures of LPS mutants used in this study. Schematic LPS structures especially highlighting the core oligosaccharide portion of LPS are illustrated. Structures of the major glycoforms of the core oligosaccharide are based on the structural analysis of an *E*. *coli* K12 derivative, W3100 [66]. Each sugar or amino sugar of the core oligosaccharide is shown by a green (Hep), violet (Gal), grey (Kdo) or blue (Glc). Phosphate groups on modified sugars are shown by red circles. Hep: L-*glycero*-D-*manno*-heptose, Kdo: 3-deoxy-D-*manno-*oct-2- ulosonic acid, GlcN: *N-*acetylglucosamine, Glc: glucose, Gal: galactose.

Previous studies have shown that various AMPs including Cap18, Cap11, Melittin and Indolicidin have LPS binding properties [21][22][23][24][25]. To investigate the potential role of LPS involved in the mechanism in more detail, the antimicrobial activity of Cap18, Cap11, Cap11-1-18m^2^, Cecropin P1, Cecropin B, Indolicidin, Melittin and Sub5 was investigated in a series of LPS mutants compared with their parental strains, BW25113, an *E*. *coli* K12 strain, and the reference strain ATCC25922. In this study, the *rfaC*, *rfaE*, *rfaF and rfaG* genes were selected for mutation (Table 7, Fig 1). These genes are involved in the synthesis and assembly of the core oligosaccharide, the middle part of the LPS molecule connecting the lipid A and the O-antigen. Except for Cap11, all the tested AMPs showed higher antimicrobial activity in the BW25113 LPS mutants compared to the parental strain BW25113 (Table 8). The highest increase (4-8x) in antimicrobial activity was detected for the AMP Melittin in all the BW25113 mutant strains. Cecropin P1 and Cecropin B is 4-8x more active in the BW25113*ΔrfaF* mutant, 2-4x more active in the BW25113*ΔrfaC* and BW25113*ΔrfaF* mutants, up to 2x fold more active in the BW25113*ΔrfaG* mutant compared to the wild-type BW25113. Sub5 showed a 2-4x increased antimicrobial activity, Cap11-1-18m^2^ and Indolicidin a 2x increased and Cap18 up to 2x increased antimicrobial activity in all BW25113 LPS mutants. Similar results were obtained for the LPS mutants in the *E*. *coli* reference strain ATCC25922. The highest increase in antimicrobial activity was observed for Melittin in the *ΔrfaC*, *ΔrfaE* and *ΔrfaF* mutant strains in which the antimicrobial activity is 8x higher compared to the ATCC25922 wild-type. A 4-fold increase in antimicrobial activity was measured for Melittin in the ATCC25922*ΔrfaG* mutant, for Cap11 and Cecropin P1 in the *ΔrfaC*, *ΔrfaE* and *ΔrfaF* mutant strain, for Cecropin B in the *ΔrfaC* and *ΔrfaE* mutant strains and for Sub5 in all tested ATCC25922 LPS mutants. Cap18, Cap11-1-18m^2^ and Indolicidin showed a 2-fold increase in activity in all the tested ATCC25922 LPS mutants, Cap11, Cecropin P1 and Cecropin B were 2-fold more active in the ATCC25922*ΔrfaG* background. Summarizing, LPS plays a central role in protecting against the antimicrobial activity of the tested AMPs, which all showed higher antimicrobial activity in LPS defective mutants.

**Table 7:** Function and Phenotype of the LPS genes selected in this study.

Gene Name	Alternative Gene Name(s)	Function in core oligosaccharide assembly	Character of the LPS core	Reference
*waaC*	*rfaC*	LPS heptosyltransferase I (HepI). Adds the first heptose sugar onto the Kdo_2_ moiety.	Heptoseless	[61],[62]
*waaE*	*rfaE*, *hldE*	Heptose 7-phosphate kinase/heptose 1-P adenyltransferase	Heptoseless	[63][64]
*waaF*	*rfaF*	LPS heptosyltransferase II (HepII). Transfers the second heptose sugar onto the heptosyl-Kdo_2_ moiety.	Kdo_2_ with one heptose	[61]
*waaG*	*rfaG*	LPS glycosyltransferase I. Add the first glucose to the outer core oligosaccharide	Intact 3 heptose, but outer core-less. Reduced phosphorylation of the inner core	[65][57]

**Table 8:** Antimicrobial activity of selected AMPs in different LPS backgrounds.

Strain								
	Antimicrobial Activity MIC [μg/ml]							
	Cap18	Cap11	Cap11-1-18m2	Cecropin P1	Cecropin B	Indolicidin	Melittin	Sub5
BW25113 wild-type	4–8	8	16	16–32	16–32	32	16–32	4–8
BW25113 Δ*rfaC*	4	8	8	8	8	16	4	2
BW25113 Δ*rfaE*	4	8	8	8	8	16	4	2
BW25113 Δ*rfaF*	4	8	8	4	4	16	4	2
BW25113 Δ*rfaG*	4	8	8	16	16	16	4	2
ATCC25922 wild-type	4	16	32	32	32	32	16	4
ATCC25922 Δ*rfaC*	2	4	16	8	8	16	2	1
ATCC25922 Δ*rfaE*	2	4	16	8	8	16	2	1
ATCC25922 Δ*rfaF*	2	4	16	8	16	16	2	1
ATCC25922 Δ*rfaG*	2	8	16	16	16	16	4	1

Data are collected as minimal inhibitory concentrations (MICs) according to the Clinical and Laboratory Standards Institute (CLSI) and expressed in μml. All MIC determinations were carried out in triplicates.

## Discussion

The number of antibiotic resistant pathogens is increasing and the capacity of currently available antimicrobial compounds to control bacterial infections is diminishing. Antimicrobial peptides are an alternative to classical antibiotics to control and fight bacterial infections. However, the lack of standardized protocols and comprehensive overviews comparing the antimicrobial activity, cytotoxicity and stability of known AMPs from different sources is one of the major challenges in identifying AMPs for further development into practical use. Very often direct comparison of the antimicrobial activity of different AMPs from different publications is not possible due to different assay conditions and different peptide purities. This study, comparing AMPs from different origins using the same assay conditions, allows a direct comparison of the antimicrobial activity, hemolytic activity, and stability of a selection of AMPs against a broader range of bacteria. In this study, we demonstrate that Cap18 and Cap11, both members of the cathelidicin family, displayed a potent efficacy in particular against Gram-negative pathogens, including pathogens found in fish and poultry production. Direct comparison of MIC values obtained in this study with previously published results is not feasible due to different assays, different strains, and different growth conditions [26][27][28][29][21][30][31][32][33][34][35][36][37]. In S2 Table we have, however, compared our results to previously reported antimicrobial activities of the selected peptides. Our results confirm the anti-Gram-negative activity of Cap18, Cap11, and Cap11-1-18m^2^, previously reported against *E*. *coli*. Our data extend the range of Gram-negatives investigated for these peptides. For Myxinidin and Pyrrhocoricin our data contradicts previously published results [20,38,39]. One possible explanation for the different results of Pyrrhocoricin could be attributed to the fact that Cudic et al. used the C-terminal amidated form of Pyrrhocoricin and Cocianich et al. used Pyrrhocoricin directly isolated from *Pyrrhocoris apterus*. For the other peptides we see a mixture of confirmations and contradictions in situations where the combination of peptide and microorganism has been investigated previously. Our analysis allow this set of peptides to be compared under identical conditions against a broad range of microorganisms, and thereby to select the potentially most suited candidate for development into an antibacterial product.

Besides a high antimicrobial activity, low cytotoxicity is a desirable characteristic for AMPs as potential drug candidates. In general, AMPs are binding to the bacterial surface by electrostatic interactions. However, some types of AMPs are able to interact not only with the bacterial surface, but also with the host cells which leads to cell lysis. Very often, the toxicity of AMPs against eukaryotic cells is one of the major obstacles for their clinical application. Melittin has a strong hemolytic activity and is a prototype of AMPs which are inducing pores, which is in agreement with previous findings [40]. Only Melittin, Cap11-1-18m^2^, Indolicidin, and Cap11 showed hemolytic activity; Cap11 only showed very low hemolytic activity. Using those peptides as drug candidates would need further modifications reducing the hemolytic activity and increasing the specificity for bacterial cells. Several different approaches to improve the specificity are described in literature. Previous studies, show that changing the net charge and reducing the hydrophobicity by introducing hydrophilic amino acids is leading to a decreased hemolytic activity [41][42][43]. Other studies have demonstrated that the introduction of D-amino acids can lower the hemolytic activity of α-helical AMPs [44]. Cap18 displayed no hemolysis in our assay using horse erythrocytes. The absence of cytotoxicity should however be expanded to other relevant species of erythrocytes before Cap18 can safely be developed into products for specific applications.

A challenge with cationic AMPs in various therapeutic contexts is its susceptibility to proteolytic degradation. Many bacteria have developed proteases, e.g. elastase in *Pseudomonas aeruginosa*, aureolysin and V8 in *Staphylococcus aureus*, leading to fast degradation of the AMPs. Moreover, gastrointestinal digestive enzymes, such as pepsin and trypsin, and proteases in the serum contribute to the low proteolytic stability of the majority of AMPs. Our data show that Cecropin P1, Cecropin B, Melittin and Indolicidin are highly sensitive to proteolytic degradation by trypsin or proteinase K. Cap18, Cap11, Cap11-1-18m^2^ and Sub5 are more stable towards proteinase K digestion. However, only Cap11, Cap11-1-18m^2^ and Sub5 showed partial stability towards trypsin digestion. In summary, these findings corroborate that especially antimicrobial peptides which have a cationic character will show fast degradation. Recent research focused on finding ways to improve proteolytic stability of cationic antimicrobial peptides. Similar strategies which can be used to reduce cytotoxicity are showing potential to reduce protease susceptibility. The incorporation of non-natural amino acids such as D-amino acids or amino acid derivate of arginine, the modification of the terminal regions including acetylation, amidation and hydrophobic tagging and the use of non-peptidic backbones (peptidomimetics) have been shown to improve the protease stability of AMPs [45] [46][47][42][48].

AMPs to be used as feed ingredients should preferably be heat stable, as feed processing usually includes a pelleting process involving temperatures between 50°C and 90°C [49]. In order to reduce the risk of spreading microbial contaminants with the feed the higher end of the temperature range is recommended [50]. Feed enzymes will either have to be applied after the pelleting process or the enzyme need to optimized for thermal stability [51]. In contrast to enzymes all the tested AMPs are highly thermostable.

The mode of action of AMPs largely depends on the bacterial cell surface and the amino acid composition of the peptide itself. According to a previously described model for the mode action, AMPs are initially attracted to the bacterial surface most likely by electrostatic bonding between the cationic peptide and the bacterial surface. Cationic peptides are likely first attracted by negatively charged lipopolysaccharide molecules in Gram-negative bacteria [8]. The negatively charged Lipopolysaccharide molecule (LPS) is one of major molecular components of the outer membrane in Gram-negative bacteria. LPS consist of three distinct components: LipidA acts as hydrophobic anchor in the outer membrane. The core oligosaccharide (core OS) composed of different sugar molecules connects the lipid A with the O-antigen, a structurally variable polysaccharide made up of repeating oligomeric units (Fig 1). In addition, bacterial membranes are primarily composed of negatively charged lipids including phosphatidylglycerol, cardiolipin and the zwitter ionic phosphatidylethanolamine. Even though the negatively charged LPS molecules are important for initial attraction of cationic peptides to the membrane, the outer membrane itself also acts as an effective permeability barrier against various harmful agents, including hydrophobic antibiotics [52][8][53][54]. In particular, mutants in the core oligosaccharide of the LPS have been shown to be more susceptible to hydrophobic agents. *E*. *coli* and *S*. Typhimurium strains that lack heptose in the LPS show a deep rough phenotype which is characterized by a reduction in the outer membrane protein content and increased sensitivity towards detergents and hydrophobic antibiotics [55][56]. An *E*. *coli* F540 *rfaG* mutant which is defective in the inner core of the LPS molecule, has a destabilized outer membrane and exhibits a 80% reduction of heptose phosphorylation which leads to an increased susceptibility towards SDS and Novobiocin [57]. Our data clearly indicates that LPS in *E*. *coli* not only acts in the attraction and attachment of antimicrobial peptides to the outer membrane, but also functions as a protection barrier against cationic AMPs, very similar to antibiotics. The mutants defective in the core LPS showed increased susceptibility to the majority of the tested AMPs. The Δ*rfaC* and Δ*rfaE* mutants, which are supposed to contain a core without heptose in *E*. *coli* K12 BW25113 and the clinical isolate ATCC25922, showed increased susceptibility towards Cap18, Cap11-1-18m^2^, Cecropin P1, Cecropin B, Indolicidin, Melittin and Sub5. Similarly, both Δ*rfaF* mutants, having a core oligosaccharide consisting of only one heptose, were more susceptible for Cap18, Cap11-1-18m^2^, Indolicidin, Melitin and Sub5. A slight difference in susceptibility was measured for Cecropin P1 and Cecropin B in the different *E*. *coli* backgrounds. Cecropin P1 and Cecropin B showed increased antimicrobial activity in BW25113Δ*rfaF* background compared to ATCC25922Δ*rfaF* and both Δ*rfaC* and Δ*rfaE* mutants. More distinct differences in susceptibility were measured in *rfaG* mutant background, which has an intact inner part of the core oligosaccharide and is only missing a functional outer part of the core oligosaccharide. The biggest differences in susceptibility between the two *E*. *coli* strains, the K12 derivative BW25113 and the clinical isolate ATCC25922, were measured for Cap11 in all tested LPS mutants. The antimicrobial susceptibility of Cap11 in the LPS mutants was identical to the wild-type BW25113 strain. In contrast, the antimicrobial activity was increased by factor 4 in the Δ*rfaC*,Δ*rfaE*,Δ*rfaF* and by factor 2 in the Δ*rfaG* mutants compared to the wild-type ATCC25922. These data suggest that the different degrees of susceptibility are depending on one hand on the character of the LPS mutation and on the other hand on the nature of the AMP. Interestingly, LPS mutants lacking heptose completely and as consequence also lacking all the negatively charged phosphate groups, showed a higher susceptibility to the majority of the tested AMPs. This indicates that LPS is not essential for antimicrobial activity of cationic peptides, even though in previous models LPS are regarded as needed for attraction and attachment via electrostatic bonding. In addition, the O-antigen does not seem to have an obvious function in the mode of action for the tested cationic AMPs since the susceptibility of all tested AMPs in *E*. *coli* K12 BW25113, which is missing the O-antigen, and the clinical isolate ATCC25922 belonging to the serotype O6 are the same, except for Cap11 and Cap11-1-18m^2^ which show only a very minor difference.

In summary, Cap18, isolated from rabbit neutrophils, is of all the tested AMPs the most active and has the highest antimicrobial activity in particular against Gram-negative foodborne pathogens. Cap18 also showed very low toxicity to horse erythrocytes and was stable at high temperatures. All these characteristics indicates that Cap18 has potential for further development as e.g. food and feed ingredient against infections caused by Gram-negative foodborne pathogens. However, Cap18 is sensitive to trypsin and proteinase K *in vitro* and further improvement addressing protease stability will be needed. In addition, our results indicate that the LPS do not play a central role in the mechanism of cationic AMPs in Gram-negative bacteria. Our findings indicate that LPS is not important in the attraction of cationic peptides to the bacterial surface of Gram-negative bacteria, but in fact acts as a protection barrier. However, other factors than LPS might also be involved in the mode of action, since various cationic AMPs show antimicrobial activity against Gram-positive bacteria which are lacking LPS.

## Supporting Information

S1 Table Primers used for creating and checking knock-out mutants. (DOC)

S2 Table Comparison of reported antimicrobial activity of selected AMPs. (DOCX)
