# Supplementary material for: Comparative Evaluation of the Antimicrobial Activity of Different Antimicrobial Peptides against a Range of Pathogenic Bacteria
Source: PLoS One. 2015 Dec 11;10(12):e0144611. doi: 10.1371/journal.pone.0144611 (PMC4684357; doi:10.1371/journal.pone.0144611)
Supplement: S1 Table — (DOC) [file pone.0144611.s001.doc]

**S1 Table. Primers used for creating and checking knock-out mutants.**

Boldface indicates regions binding to pKD4 for amplification of the kanamycin resistance gene.

|  | Primer | Sequence |
| --- | --- | --- |
| *Used for creating knock-outs* |  |  |
| *rfaC* forward | 1180 | 5´- ATTTCAAAATTATCTATTAAAGAGTTGTAATTTAAATCAT**GTGTAGGCTGGAGCTGCTTC** - 3´ |
| *rfaC* reverse | 1181 | 5´- GAACTCAACGCGCTATTGTTACAAGAGGAAGCCTGACGG**CATATGAATATCCTCCTTAG** - 3´  -3’ |
| *rfaE* forward | 1182 | 5´- TATTATCGCGCGCAAATTTTGAATCTCTCAGGAGACAGGA**GTGTAGGCTGGAGCTGCTTC** - 3´  -3 |
| *rfaE* reverse | 1183 | 5´- CCTGCCTGCTACGAAGCGAGATCTGTGAACCGCTTTCCAG**CATATGAATATCCTCCTTAG** - 3´  -3’ |
| *rfaF* forward | 1184 | 5´- CGCCCATCGACGATGTTTTAACGATCAGAACCCGCATCCG**GTGTAGGCTGGAGCTGCTTC** - 3´  -3´ |
| *rfaF* reverse | 1185 | 5´- ATACATGGCCTGGCTGAATCGTGACGCATAAGAGCTCTGC**CATATGAATATCCTCCTTAG** - 3´  -3’ |
| *rfaG* forward | 1186 | 5´- TACTTCCCTCCTCCACGACAGGTACGTCGTTATGATCGTT**GTGTAGGCTGGAGCTGCTTC** - 3´  -3´ |
| *rfaG* reverse | 1187 | 5´- TAACGTGGCAAACGGCTCTTTAAGTTCAACCATCCAGACC**CATATGAATATCCTCCTTAG** - 3´  -3’ |
|  |  |  |
| *Used for checking mutants* |  |  |
| *rfaC* forward | 1210 | 5´- CAATCGGACCAATGTTTATTAGA -3´  -3´ |
| *rfaC* reverse | 1201 | 5´- GAACTCAACGCGCTATTGTTACAAG -3´  -3´ |
| *rfaE* forward | 1202 | 5´- TATTATCGCGCGCAAATTTTGAATC -3´ |
| *rfaE* reverse | 1203 | 5´- CCTGCCTGCTACGAAGCGAGATCTG -3´ |
| *rfaF* forward | 1204 | 5´- CGCCCATCGACGATGTTTTAACGAT -3´  -3´ |
| *rfaF* reverse | 1205 | 5´- ATACATGGCCTGGCTGAATCGTGACG -3´  -3´ |
| *rfaG* forward | 1206 | 5´- TACTTCCCTCCTCCACGACAGGTACG -3´  -3´ |
| *rfaG* reverse | 1207 | 5´- TAACGTGGCAAACGGCTCTTTAAGTT -3´  -3´ |
