# Supplementary material for: Comparative Evaluation of the Antimicrobial Activity of Different Antimicrobial Peptides against a Range of Pathogenic Bacteria
Source: PLoS One. 2015 Dec 11;10(12):e0144611. doi: 10.1371/journal.pone.0144611 (PMC4684357; doi:10.1371/journal.pone.0144611)
Supplement: S2 Table — (DOCX) [file pone.0144611.s002.docx]

**S2 Table. Comparison of reported antimicrobial activity of selected AMPs**

|  | Cap18 | | | Cap11 | | | Cap11-1-18m^2^ | | | Cecropin B | | |
| --- | --- | --- | --- | --- | --- | --- | --- | --- | --- | --- | --- | --- |
|  | literature data | | This study | literature data | | This study  MIC | literature data | | This study  MIC | literature data | | This study  MIC |
|  | MIC | Ref | MIC | MIC | Ref |  | MIC | Ref |  | MIC | Ref |  |
| *Y. ruckeri* | - | - | 2 | - | - | 4 | - | - | 8-16 | 1.95-3.11^+^ | [1] | 32 |
| *A. salmonicida* | - | - | 2 | - | - | 4 | - | - | 32-64 | 5.06-9.73^+^ | [1] | 32-64 |
| *S. enterica* | - | - | 4 | - | - | 8 | - | - | 16 | - |  | 32 |
| *C. jejuni* | - | - | 1 | - | - | 4 | - | - | 2 | - |  | 16 |
| *E. coli* | 0.4 – 8.87^+^ | [2] | 4-8* | activity | [3] | 8-16* | activity | [3] | 16-32* | 6.62-12.85^+^ | [4] | 16-32* |
| *P. aeruginosa* | 0.22 – 2.75^+^ | [2] | 4-8**** | activity | [3] | 8**** | activity | [3] | 16-32**** | 65.01^+^ | [4] | 64**** |
|  | 2.5 | [5] |  |  |  |  |  |  |  |  |  |  |
| *S. aureus* | 1.82 – 6.03^+^  16.7 | [2]  [5] | ≥32*** | - | - | 16-32*** | - | - | 16*** | 259.66^+^ | [4] | >256*** |
| *E. faecalis* | - | - | 8** | - | - | 16-32** | - | - | 8-16** | 259.66^+^ | [4] | >256** |
| *L. monocytogenes* | - | - | 2-4 | - | - | 32 | - | - | 16 | - |  | >256 |

|  | Cecropin P1 | | | Bac2A-NH_2_ | | | Bac2A | | | Sub5-NH_2_ | | |
| --- | --- | --- | --- | --- | --- | --- | --- | --- | --- | --- | --- | --- |
|  | literature data | | This study  MIC | literature data | | This study  MIC | literature data | | This study  MIC | literature data | | This study  MIC |
|  | MIC | Ref |  | MIC | Ref |  | MIC | Ref |  | MIC | Ref |  |
| *Y. ruckeri* | 2.1^+^ | [1] | 32 | - | - | ≥256 | - | - | 256 | - | - | 16-32 |
| *A. salmonicida* | 3.36^+^-10.50^+^ | [1] | 64 | - | - | 128 | - | - | >256 | - | - | 8 |
| *S. enterica* | 0.5 – 8 | [6] | ≥128 | 34  34.1^+^  32 | [7]  [8]  [9] | 128 | - | - | >256 | 7.92^+^ | [8] | 8 |
| *C. jejuni* | - | - | >256 | - | - | 64-128 | - | - | ≥256 | - | - | 8 |
| *E. coli* | 4*  0.25-1  13.87^+^ | [10]  [6]  [4] | 16-32* | 17  17.05^+^  4 | [7]  [8]  [9] | 64* | - | - | 256* | 3.96^+^ | [8] | 4* |
| *P. aeruginosa* | 4-64  34.88^+^ | [6]  [4] | >256**** | 50  49.73^+^  8 | [7]  [8]  [9] | 128-256*** | - | - | 256**** | 1.9^+^ | [8] | 8**** |
| *S. aureus* | 32->128  280 | [6]  [4] | >256*** | 4-16  17  17.05^+^ | [11]  [7]  [8] | 128*** | - | - | >256*** | 1.9^+^ | [8] | 8*** |
| *E. faecalis* | >128  280 | [6]  [4] | >256** | 2 **  17  17.05^+^ | [11]  [7]  [8] | 16-32** | - | - | 64** | 1.9^+^ | [8] | 4-8** |
| *L. monocytogenes* | - |  | >256 | 0.25 | [11] | 8 | - | - | 32 | - | - | 2 |

|  | Myxinidin | | | Myxinidin-NH^2^ | | | Melittin | | | Indolicidin | | |
| --- | --- | --- | --- | --- | --- | --- | --- | --- | --- | --- | --- | --- |
|  | literature data | | This study  MIC | literature data | | This study  MIC | literature data | | This study  MIC | literature data | | This study  MIC |
|  | MIC | Ref |  | MIC | Ref |  | MIC | Ref |  | MIC | Ref |  |
| *Y. ruckeri* | 2 | [12] | >256 | - | - | >256 | - | - | 16-32 | - | - | ≥64 |
| *A. salmonicida* | 2 | [12] | >256 | - | - | >256 | - | - | >64 | - | - | ≥64 |
| *S. enterica* | 2.5  26.54^+^ | [12]  [13] | >256 | - | - | >256 | 5.7^+^  16 | [14]  [15] | 32-64 | 2-32  7.62^+^ | [6]  [8] | 64 |
| *C. jejuni* | - | - | >256 | - | - | >256 |  |  | 2-4 | - | -sw | 16 |
| *E. coli* | 2  6.64^+^ | [12]  [13] | >256* | - | - | >256* | 11.1^+^  32.02^+^  5.7^+^*  8*  30*  1.79-3.56^+^* | [16]  [17]  [14]  [15]  [18]  [19] | 16* | 0.5-16  15.25^+^  31.25* | [6]  [8]  [20] | 32* |
| *P. aeruginosa* | 7-10  39.81^+^ | [12]  [13] | >256**** | - | - | >256**** | 32.02^+^  100 ****  7.12–14.23^+^**** | [17]  [18]  [19] | ≥64**** | 8-128  62.9^+^ | [6]  [8] | >64**** |
| *S. aureus* | 13.27^+^ | [13] | >256*** | - | - | >256*** | 10.25^+^  15.94^+^  22.77^+^***  3.56-7.12^+^*** | [16]  [17]  [14]  [19] | 2-4*** | 4-32  30.5^+^  8  31.25 ^+^ | [6]  [8]  [11]  [20] | 32*** |
| *E. faecalis* |  |  | >256** | - | - | >256** | 1-8  1.79-3.56^+^** | [21]  [19] | 2-4** | 32->128  30.5^+^  >64** | [6]  [8]  [11] | 32** |
| *L. monocytogenes* |  |  | >256 | - | - | >256 | 17.93^+^  11.38^+^ | [22]  [23] | 2-4 | 4 | [11] | 4 |

|  | Pyrrhocoricin | | | Apidaecin IA | | | Metalnikowin I | | |
| --- | --- | --- | --- | --- | --- | --- | --- | --- | --- |
|  | literature data | | This study^2)^  MIC | literature data | | This study  MIC | literature data | | This study  MIC |
|  | MIC | Ref |  | MIC | Ref |  | MIC | Ref |  |
| *Y. ruckeri* | - | - | >256^2)^ | - | - | 256 | - | - | >256 |
| *A. salmonicida* | - | - | >256^2)^ | - | - | >256 | - | - | >256 |
| *S. enterica* | >23.41^+1)^ | [24] | >256^2)^ | 0.1 | [25] | 64 | - | - | >256 |
|  | 0.5 -3^3)^ | [26] |  |  |  |  |  |  |  |
| *C. jejuni* | - | - | - | - | - | - | - | - | - |
| *E. coli* | 11.70-23.41^+1)^ | [24] | >256*^2)^ | 0.1 | [25] | 32* | - | - | >256* |
|  | 0.5-1^3)^ | [26] |  |  |  |  |  |  |  |
| *P. aeruginosa* | 11.70-23.41^+1)^ | [24] | >256****^2)^ | - | - | >256**** | - | - | >256**** |
| *S. aureus* | >23.41^+1)^ | [24] | >256***^2)^ | - | - | >256*** | - | - | >256*** |
|  | >50^3)^ | [26] |  |  |  |  |  |  |  |
| *E. faecalis* | >40^3)^ | [26] | >256**^2)^ | - | - | >256** | - | - | >256** |
| *L. monocytogenes* | - | - | >256^2)^ | - | - | >256 | - | - | >256 |

* *Escherichia coli* ATCC25922, ** *Enterococcus faecalis* ATCC29212, *** *Staphylococcus aureu*s ATCC29213, **** *Pseudomonas aeruginosa* ATCC27853

1) Pyrrhocoricin isolated from *Pyrrhocoris apterus*, 2) Synthetic Pyrrhocoricin, no N-terminal amidation, 3) Synthetic Pyrrhocoricin-NH_2_

All MIC values are given in μg/ml. MIC data which was converted from μM to μg/ml is indicated with ^+^

S2 Table References

1. Kjuul AK, Büllesbach EE, Espelid S, Dunham R, Jørgensen TÒ, Warr GW, et al. Effects of cecropin peptides on bacteria pathogenic to fish. J Fish Dis. 1999;22: 387–394. doi:10.1046/j.1365-2761.1999.00191.x

2. Travis SM, Anderson NN, Forsyth WR, Espiritu C, Conway BD, Greenberg EP, et al. Bactericidal Activity of Mammalian Cathelicidin-Derived Peptides. Infect Immun. 2000;68: 2748–2755. doi:10.1128/IAI.68.5.2748-2755.2000

3. Okuda D, Yomogida S, Kuwahara-arai K. Augmentation of the antimicrobial activities of guinea pig cathelicidin CAP11-derived peptides by amino acid substitutions. 2009; 501–508. doi:10.3892/ijmm

4. Moore a J, Beazley WD, Bibby MC, Devine D a. Antimicrobial activity of cecropins. J Antimicrob Chemother. 1996;37: 1077–1089. Available: http://www.ncbi.nlm.nih.gov/pubmed/8836811

5. Brogden KA, Kalfa VC, Ackermann MR, Palmquist DE, McCray PB, Tack BF. The ovine cathelicidin SMAP29 kills ovine respiratory pathogens in vitro and in an ovine model of pulmonary infection. Antimicrob Agents Chemother. 2001;45: 331–334. doi:10.1128/AAC.45.1.331-334.2001

6. Giacometti A, Cirioni O, Greganti G, Quarta M, Scalise G. In vitro activities of membrane-active peptides against gram-positive and gram-negative aerobic bacteria. Antimicrob Agents Chemother. 1998;42: 3320–3324.

7. Hilpert K, Volkmer-Engert R, Walter T, Hancock REW. High-throughput generation of small antibacterial peptides with improved activity. Nat Biotechnol. 2005;23: 1008–1012. doi:10.1038/nbt1113

8. Mania D, Hilpert K, Ruden S, Fischer R, Takeshita N. Screening for antifungal peptides and their modes of action in Aspergillus nidulans. Appl Environ Microbiol. 2010;76: 7102–7108. doi:10.1128/AEM.01560-10

9. Wu M, Hancock RE. Improved derivatives of bactenecin, a cyclic dodecameric antimicrobial cationic peptide. Antimicrob Agents Chemother. 1999;43: 1274–6. Available: http://www.ncbi.nlm.nih.gov/pubmed/10223951

10. Haukland HH, Ulvatne H, Sandvik K, Vorland LH. The antimicrobial peptides lactoferricin B and magainin 2 cross over the bacterial cytoplasmic membrane and reside in the cytoplasm. FEBS Lett. 2001;508: 389–393. doi:10.1016/S0014-5793(01)03100-3

11. Friedrich CL, Moyles D, Beveridge TJ, Hancock RE. Antibacterial action of structurally diverse cationic peptides on gram-positive bacteria. Antimicrob Agents Chemother. 2000;44: 2086–2092. doi:10.1128/AAC.44.8.2086-2092.2000

12. Subramanian S, Ross NW, MacKinnon SL. Myxinidin, a novel antimicrobial peptide from the epidermal mucus of hagfish, Myxine glutinosa L. Mar Biotechnol (NY). 2009;11: 748–757. doi:10.1007/s10126-009-9189-y

13. Cantisani M, Leone M, Mignogna E, Kampanaraki K, Falanga A, Morelli G, et al. Structure-activity relations of myxinidin, an antibacterial peptide derived from the epidermal mucus of hagfish. Antimicrob Agents Chemother. 2013;57: 5665–5673. doi:10.1128/AAC.01341-13

14. Lv Y, Wang J, Gao H, Wang Z, Dong N, Ma Q, et al. Antimicrobial properties and membrane-active mechanism of a potential α-helical antimicrobial derived from cathelicidin PMAP-36. PLoS One. 2014;9: e86364. doi:10.1371/journal.pone.0086364

15. Dong N, Ma Q, Shan A, Lv Y, Hu W, Gu Y, et al. Strand length-dependent antimicrobial activity and membrane-active mechanism of arginine- and valine-rich β-hairpin-like antimicrobial peptides. Antimicrob Agents Chemother. 2012;56: 2994–3003. doi:10.1128/AAC.06327-11

16. Asthana N, Yadav SP, Ghosh JK. Dissection of antibacterial and toxic activity of melittin: a leucine zipper motif plays a crucial role in determining its hemolytic activity but not antibacterial activity. J Biol Chem. 2004;279: 55042–55050. doi:10.1074/jbc.M408881200

17. Saravanan R, Bhunia A, Bhattacharjya S. Micelle-bound structures and dynamics of the hinge deleted analog of melittin and its diastereomer: Implications in cell selective lysis by d-amino acid containing antimicrobial peptides. Biochim Biophys Acta - Biomembr. 2010;1798: 128–139. doi:10.1016/j.bbamem.2009.07.014

18. Al-Ani I, Zimmermann S, Reichling J, Wink M. Pharmacological synergism of bee venom and melittin with antibiotics and plant secondary metabolites against multi-drug resistant microbial pathogens. Phytomedicine. 2015;22: 245–255. doi:10.1016/j.phymed.2014.11.019

19. Kuhn-Nentwig L, Müller J, Schaller J, Walz A, Dathe M, Nentwig W. Cupiennin 1, a new family of highly basic antimicrobial peptides in the venom of the spider Cupiennius salei (Ctenidae). J Biol Chem. 2002;277: 11208–11216. doi:10.1074/jbc.M111099200

20. Jindal HM, Le CF, Mohd Yusof MY, Velayuthan RD, Lee VS, Zain SM, et al. Antimicrobial Activity of Novel Synthetic Peptides Derived from Indolicidin and Ranalexin against Streptococcus pneumoniae. PLoS One. 2015;10: e0128532. doi:10.1371/journal.pone.0128532

21. Mataraci E, Dosler S. In vitro activities of antibiotics and antimicrobial cationic peptides alone and in combination against methicillin-resistant Staphylococcus aureus biofilms. Antimicrob Agents Chemother. 2012;56: 6366–6371. doi:10.1128/AAC.01180-12

22. Moerman L, Bosteels S, Noppe W, Willems J, Clynen E, Schoofs L, et al. Antibacterial and antifungal properties of alpha-helical, cationic peptides in the venom of scorpions from southern Africa. Eur J Biochem. 2002;269: 4799–4810. doi:10.1046/j.1432-1033.2002.03177.x

23. Park SC, Kim JY, Jeong C, Yoo S, Hahm KS, Park Y. A plausible mode of action of pseudin-2, an antimicrobial peptide from Pseudis paradoxa. Biochim Biophys Acta - Biomembr. 2011;1808: 171–182. doi:10.1016/j.bbamem.2010.08.023

24. Cociancich S, Dupont A, Hegy G, Lanot R, Holder F, Hetru C, et al. Novel inducible antibacterial peptides from a hemipteran insect, the sap-sucking bug Pyrrhocoris apterus. Biochem J. 1994;300 ( Pt 2: 567–75. Available: http://www.ncbi.nlm.nih.gov/pubmed/8002963

25. Casteels P, Ampe C, Jacobs F, Vaeck M, Tempst P. Apidaecins: antibacterial peptides from honeybees. EMBO J. 1989;8: 2387–2391.

26. Cudic M, Condie B a, Weiner DJ, Lysenko ES, Xiang ZQ, Insug O, et al. Development of novel antibacterial peptides that kill resistant isolates. Peptides. 2002;23: 2071–2083. Available: http://www.ncbi.nlm.nih.gov/pubmed/12535685
